# Influence of molecular imaging on patient selection for treatment intensification prior to salvage radiation therapy for prostate cancer: a post hoc analysis of the PROPS trial

**DOI:** 10.1186/s40644-023-00570-x

**Published:** 2023-06-08

**Authors:** Samuel Tremblay, Mofarej Alhogbani, Andrew Weickhardt, Ian D Davis, Andrew M Scott, Rodney J Hicks, Ur Metser, Sue Chua, Reena Davda, Shonit Punwani, Heather Payne, Nina Tunariu, Bao Ho, Sympascho Young, Mahukpe Narcisse Ulrich Singbo, Glenn Bauman, Louise Emmett, Frédéric Pouliot

**Affiliations:** 1 CHU de Québec and Université Laval, Québec, Québec Canada; 2 Austin Health and University of Melbourne, Olivia Newton-John Cancer Research Institute, La Trobe University, Melbourne, Australia; 3 Monash University Eastern Health Clinical School, Box Hill, VIC Australia; 4 Peter MacCallum Cancer Centre, Melbourne, Australia; 5 University of Toronto, Toronto, ON Canada; 6 Royal Marsden Hospital, London, UK; 7 University College London, London, UK; 8 St. Vincent’s Hospital, Sydney, NSW Australia; 9 London Health Sciences Centre, London, ON Canada

**Keywords:** Prostate cancer, Biochemical recurrence, Molecular imaging, PSMA, Choline

## Abstract

**Background:**

The impact of molecular imaging (MI) on patient management after biochemical recurrence (BCR) following radical prostatectomy has been described in many studies. However, it is not known if MI-induced management changes are appropriate. This study aimed to determine if androgen deprivation therapy (ADT) management plan is improved by MI in patients who are candidates for salvage radiation therapy.

**Methods:**

Data were analyzed from the multicenter prospective PROPS trial evaluating PSMA/Choline PET in patients being considered for salvage radiotherapy (sRT) with BCR after prostatectomy. We compared the pre- and post-MI ADT management plans for each patient and cancer outcomes as predicted by the MSKCC nomogram. A higher percentage of predicted BCR associated with ADT treatment intensification after MI was considered as an improvement in a patient’s management.

**Results:**

Seventy-three patients with a median PSA of 0.38 ng/mL were included. In bivariate analysis, a positive finding on MI (local or metastatic) was associated with decision to use ADT with an odds ratio of 3.67 (95% CI, 1.25 to 10.71; p = 0.02). No factor included in the nomogram was associated with decision to use ADT. Also, MI improved selection of patients to receive ADT based on predicted BCR after sRT : the predicted nomogram 5-year biochemical-free survivals were 52.5% and 43.3%, (mean difference, 9.2%; 95% CI 0.8 to 17.6; p = 0.03) for sRT alone and ADT±sRT subgroups, while there was no statistically significant difference between subgroups before MI.

**Conclusions:**

PSMA and/or Choline PET/CT before sRT can potentially improve patient ADT management by directing clinicians towards more appropriate intensification.

**Supplementary Information:**

The online version contains supplementary material available at 10.1186/s40644-023-00570-x.

## Introduction

Prostate cancer (PCa) is the second most frequently diagnosed cancer and the fifth cause of cancer death in men worldwide [1]. Primary PCa locoregional therapies are mainly radical prostatectomy (RP) and radiotherapy. Unfortunately, biochemical recurrence (BCR) can occur in up to 40% after 10-year following RP [2, 3]. Following BCR, conventional imaging such as computed tomography (CT) and bone scintigraphy lack sensitivity for the detection of local or metastatic recurrences at a low level of prostate-specific antigen (PSA) values [4].

There is a growing role for molecular imaging (MI) targeting Prostate Specific Membrane Antigen (PSMA), Choline metabolism, or amino acid transporter using specific tracers. These novel approaches have been shown to be significantly more accurate than conventional imaging for the detection of PCa metastasis, especially after primary therapies and at low PSA values [5–8]. These new imaging modalities are now considered a standard of care for re-staging patients after RP [9]. While many studies have reported how MI can impact the management of PCa, few prospective studies reported if the change in management induced by MI improved the patient’s outcomes [10].

The PROPS trial was conducted to evaluate the detection rate of lesion by PSMA-PET/CT and/or Choline-PET/CT in patients who were considered for salvage radiotherapy (sRT) after RP and who had negative or equivocal conventional imaging [8, 11]. The PROPS trial showed that these MI technologies had an important impact on the management plan for clinicians, leading to a management change in 42% of cases [8]. However, it is unknown if these management changes improve patient care. In this study, we seek to evaluate if PSMA-PET/CT or Choline-PET/CT MI improves patient selection for treatment intensification with ADT in patients suitable for sRT, based on a predictive nomogram.

## Materials and methods

Full materials and methods of the PROPS trial have been previously published [8, 11]. Briefly, the PROPS trial is a prospective, international multicenter trial including men with features of high-risk prostate cancer being considered for sRT and who had PSA recurrence after RP. The study protocol was approved by all institutional ethics board and registered on clinicaltrials.gov (NCT02131649). Initial inclusion criteria were : patients with biopsy confirmed PCa prior RP for pT1-pT3, N0/Nx disease, a rising PSA level of at least 0.2 ng/mL (3 consecutive rises documented a minimum of 2 weeks apart), and at least one high-risk feature (PSA > 1 ng/mL, ≥ pT3b, Gleason score > 7, or PSA doubling time ≤ 10 months). All patients included had a negative or equivocal diagnostic CT and bone scan 12 weeks before enrollment and had a planned management of standard sRT with curative intent. Patients underwent PSMA-PET/CT and/or Choline-PET/CT, and pelvic multiparametric pelvic or whole-body MRI to identify potential recurrence location. All treating physicians were asked to undertake a pre-imaging questionnaire to document their intended management, including planned sRT site, doses and fractions of radiotherapy, and ADT duration if it was planned. A second questionnaire was completed after imaging to document management changes. The study did not dictate the treatments to be received, but were all documented. The study provided for three-year follow-up of patients allowing for assessment of short term clinical endpoints (such as PSA response post treatment) but not long term outcomes (such as long term biochemical control, disease free survival, metastases free survival or overall survival).

**Fig. 1:**
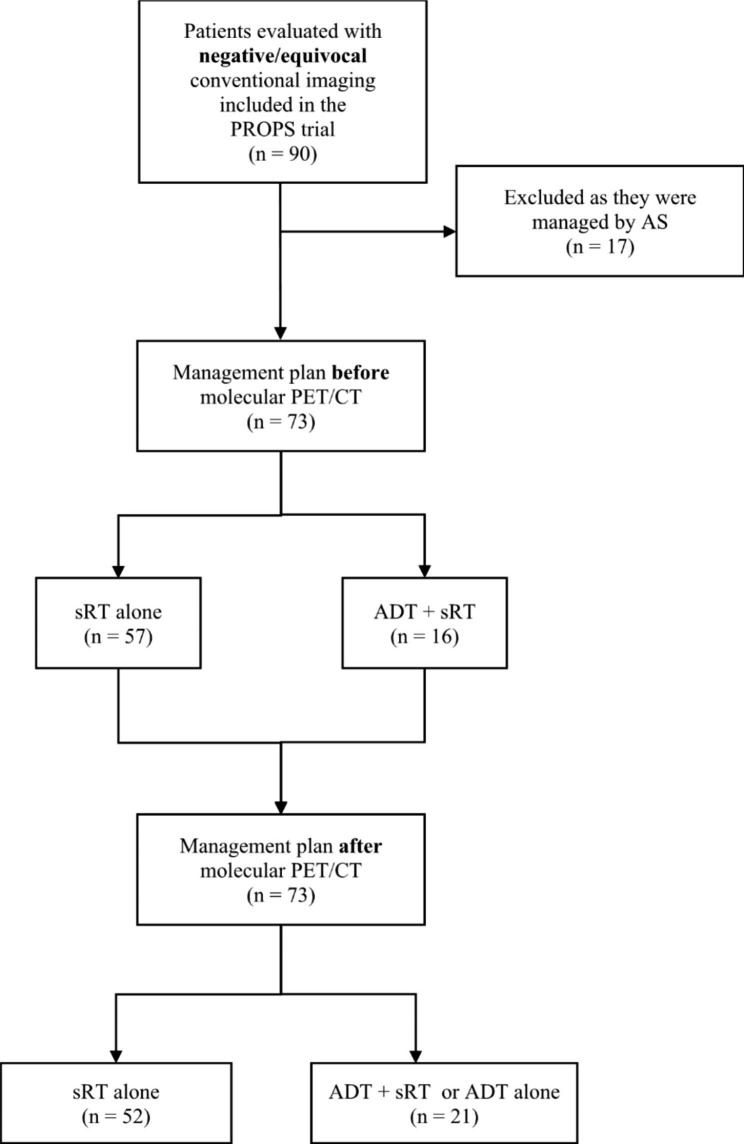
Consort diagram summarizing patients included in our analysis. Salvage radiotherapy (sRT); Androgen deprivation therapy (ADT); Active surveillance (AS).

For the current study, management plans were analyzed pre- and post-imaging for every patient initially included in the PROPS trial. Patients were evaluated based on treatment decisions before and after MI. The addition of ADT to sRT or ADT alone (ADT±sRT) was considered as a treatment intensification. Patients managed by active surveillance (AS) after new imaging modalities were excluded from final analyses because we considered that these patients could have been put on AS for aggressive (not responsive to sRT) or indolent disease (not benefiting from sRT). A detailed consort diagram summarizing included patients for this study is shown in Fig. 1. As a proxy for long term outcomes, the updated Stephenson et al. predictive nomogram for sRT after RP was used to evaluate the predicted 5- and 10-year biochemical-free survival according to patient clinicopathological characteristics [12]. Criteria for the nomogram were: surgical Gleason Grade (≤ 6, 7, 8, 9–10), extraprostatic extension (Yes/No), surgical margin status (Positive/Negative), seminal vesicle invasion (Yes/No), pre-sRT PSA level (ng/mL) and prostate bed radiation dose (≥ 6600 or < 6600 cGy) and use of ADT with sRT. Projected outcome for every patient was calculated assuming sRT alone (no neoadjuvant or concurrent ADT) as the baseline (pre-MI) treatment plan.

The primary endpoint of this study was the percentage of BCR predicted by the Stephenson nomogram before and after MI. A higher percentage of BCR associated with treatment intensification (ADT±sRT) after MI was considered as improved management.

### Statistical analysis

Analytical statistics were generated by SAS 9.4 Software. Bivariate logistic regression with Wald-based confidence intervals was used to correlate the association between imaging findings, Stephenson’s criteria and treatment decision. The comparison of mean predicted outcomes were made using two-sample Student t-tests. All tests were 2-sided, and a *p*-value of 0.05 or less was considered statistically significant.

## Results

Ninety patients were included in the PROPS Trial and the management plan was changed by the treating physician in 42% of patients (38/90) after re-imaging by PSMA PET/CT and/or choline PET/CT. Of the 38 patients that had a change in management after MI, 6 (7%) had ADT de-intensification from ADT + sRT to sRT alone, 15 patients (17%) had ADT intensification (prescription of ADT alone or in combination to sRT from either pre-imaging surveillance or sRT alone) and 17 (19%) had ADT de-intensification to surveillance and were excluded from final analyses. All patients were imaged with Choline PET/CT, and 23 patients (32%) were also imaged with PSMA PET/CT. Patient characteristics are summarized in Table 1.

**Table 1:** Patients characteristics

Characteristics	Value
Number of patients	*N* = 73
Surgical Gleason score no. (%)	
• 6 • 7 • 8 • 9	1 (1.4) 52 (71.2) 11 (15.1) 9 (12.13)
Positive surgical margins no. (%)	45 (61.6)
Extracapsular Extension no. (%)	25 (34.2)
Seminal Vesicle Invasion no. (%)	16 (21.9)
Pre-sRT PSA Level ng/ml median. (range)	0.38 (0.29–0.93)

We first assessed if MI influenced clinicians in the decision to intensify therapy. We found that a positive finding on MI, whether local or metastatic, was associated with a decision to prescribe ADT (Table 2, odds ratio = 3.67; 95% CI, 1.25 to 10.71; p = 0.02). If a metastatic lesion was found on MI, the odds ratio increased to 3.83 (95% CI, 1.11 to 13.30; p = 0.03). Interestingly, none of the Stephenson nomogram criteria used separately was associated with a decision to prescribe ADT based on the initial study cohort (Table 3). Patient’s characteristics from the initial study are summarized in the supplementary Table 1.

**Table 2:** Association between imaging findings location with PSMA- and/or Choline-PET/CT and treatment decision. Salvage radiotherapy (sRT); Androgen deprivation therapy (ADT).

		Therapeutic decision					
		sRT		ADT ± sRT			
	Location	n/N	%	n/N	%	Odds Ratio (95% CI)	P-value^1^
**Molecular imaging findings**	Extraprostatic Fossa Negative	6/13 6/10 40/50	46.2 60.0 80.0	7/13 4/10 10/50	53.8 40.0 20.0	4.67(1.28;16.99) 2.67(0.63;11.28)	0.0490*
	Positive Negative	12/23 40/50	52.2 80.0	11/23 10/50	47.8 20.0	3.67(1.25;10.71)	0.0175*
	Metastatic Non-metastatic	6/13 46/60	46.2 76.7	7/13 14/60	53.8 23.3	3.83(1.11;13.30)	0.0342*

^1^Based on Bivariate Logistic Regression with Wald confidence interval, and Firth Correction for bias (C) when

appropriate. * <0.05

**Table 3:** Stephenson nomogram criteria as predictors of therapeutic change after molecular imaging based on the whole study cohort. Salvage radiotherapy (sRT); Androgen deprivation therapy (ADT).

	Therapeutic decision	n	Mean	SD	95% CI	Odds Ratio (95% CI)	P-value^1^
**Pre-sRT PSA**	sRT ADT ± sRT	71 19	0.39 0.63	0.49 0.50	(0.28; 0.51) (0.39; 0.87)	2.63(0.92;7.50)	0.0699^T^
**Surgical Gleason score**	sRT ADT ± sRT	71 20	7.42 7.50	0.71 0.89	(7.25; 7.59) (7.08; 7.92)	1.15(0.60;2.19)	0.6814
		**Therapeutic decision**					
		**sRT**		**ADT ± sRT**			
		**n/N**	**%**	**n/N**	**%**	**Odds Ratio** **(95% CI)**	**P-value** ^**1**^
**Extraprostatic extention**	No Yes	28/71 43/71	39.4 60.6	6/20 14/20	30.0 70.0	1.52(0.52;4.42)	0.4429
**Surgical margins**	Negatives Positives	51/71 20/71	71.8 28.2	13/20 7/20	65.0 35.0	1.37(0.48;3.94)	0.5556
**Seminal vesicle invasion**	No Yes	55/71 16/71	77.5 22.5	14/20 6/20	70.0 30.0	0.68(0.22;2.05)	0.4924

^1^Based on Bivariate Logistic Regression with Wald confidence interval, and Firth Correction for bias (C)

when appropriate. T < 0.15

To determine the appropriateness of ADT intensification or de-intensification, we raised the hypothesis that an appropriate ADT management change induced by MI would intensify ADT prescription in patients with worst nomogram predicted prognosis, and de-intensify ADT prescription in patients with better nomogram predicted prognosis. To determine the risk of recurrence for each patient, the Stephenson nomogram was used [12]. Before MI, the predicted mean 5-year biochemical-free survival was 50.9% and 46.1% (mean difference, 4.8%; 95% CI -4.6 to 14.2) for sRT alone and ADT±sRT subgroups, respectively (p = 0.3). After MI, the predicted mean 5-year biochemical-free survival was 52.5% and 43.3%, (mean difference, 9.2%; 95% CI 0.8 to 17.6) for sRT alone and ADT±sRT subgroups, respectively (p = 0.03). Indeed, the difference between the two subgroups in predicted 5-year biochemical-free survival almost doubled after MI, increasing to 9.2% from 4.8% (Supplemental Fig. 1). The predicted 10-year biochemical-free survival mean difference was also statistically significant between subgroups after MI (9.7%; 95% CI 1.9 to 18.2, p = 0.03) while it was not before (Table 4).

**Table 4:** Predicted 5- and 10-year biochemical free survival (BFS) for salvage radiotherapy (sRT) after radical prostatectomy based on the updated Stephenson nomogram

		Therapeutic decision			
		sRT alone (%)	ADT ± sRT (%)	Mean Difference (95% CI)	P-value^1^
**Pre-molecular imaging**	5-year BFS probability	50.91	46.13	4.79 (-4.63;14.20)	0.3141
	10-year BFS probability	38.16	33.56	4.60 (-4.99;14.18)	0.3423
**Post-molecular imaging**	5-year BFS probability	52.5	43.3	9.2 (0.78;17.56)	0.0327*
	10-year BFS probability	39.94	30.24	9.70 (1.93;18.22)	0.0260*

Based on two-sample Student’s t-Tests * < 0.05

## Discussion

In recent years, MI use has been increasingly adopted into clinical practice. These new imaging modalities have clearly demonstrated an increase in the accuracy for identifying metastasis when compared to conventional imaging, leading to a shift towards treatment intensification, either by targeting oligometastatic disease and/or by adding ADT. The benefits of treatment intensification in patients with recurrent PCa after RP is poorly studied and mostly based on the extrapolation of small studies performed in metastatic patients proven by conventional imaging [13, 14]. So far, no prospective muticenter study has evaluated long-term prognostic outcomes of these patients diagnosed early with MI.

With 42% management changes observed after MI, our study compares to recent literature [15–17]. Here we were able to demonstrate that a positive finding on MI prompted clinicians to intensify therapy, at least with ADT. Interestingly, clinicians tended to intensify therapy if a lesion was detected, no matter if it was in the prostate bed or as a metastasis. This means that clinicians appear to be influenced by MI findings in their therapeutic decision-making and consider both local and metastatic lesions as being more aggressive.

We also demonstrated that the therapeutic intensification induced by MI was appropriate for this cohort of patients. Using the predictive Stephenson nomogram, we modeled a statistically significant biochemical free-survival difference between patients with MI directed therapeutic intensification (ADT±sRT subgroup) management plans compared to the projected results with sRT alone at 5- and 10-years.

These results suggest that MI can differentiate patients with a poorer prognosis who might better benefit from therapeutic intensification. Therefore, MI may integrate many poor prognostic factors as an imageable lesion that guides the clinicians toward identification of more aggressive disease.

Literature on the benefits of treatment changes after MI is limited. The EMPIRE-1 study showed promising results with ^18^ F-fluciclovine-PET/CT. One hundred sixty-five patients with negative conventional imaging were randomized to sRT directed by conventional imaging alone or to conventional plus ^18^ F-fluciclovine-PET/CT. Three-year event-free survival was significantly superior to the ^18^ F-fluciclovine-PET/CT group (75.5% vs. 63.0%, *p* = 0.0028). Importantly, toxicity was similar in both groups [10]. Emmett et al. also demonstrated interesting results with PSMA-PET/CT. Two hundred sixty patients with a rising PSA level after RP were prospectively referred to PSMA-PET/CT. Three years’ freedom from progression (FFP) after sRT was highly predictive with a reported FFP of 64.5% (120/186) overall, 81% (81/100) with fossa-confined disease and 45% (39/86) with extrafossa disease after PSMA-PET/CT [18]. These results and ours are supporting that MI rationally helps to locate disease recurrence and select patients that could benefit from a treatment intensification and potentially impact oncologic outcomes. The Ongoing PATRON trial (NCT04557501) and PSMA-SRT trial (NCT03582774) are two randomized Phase III studies that will evaluate cancer outcomes of treatment intensification with PSMA PET/CT compared to conventional imaging on a 5-year perspective and will certainly help to clarify how to manage these oligometastatic patients.

There are limitations to our study. The original protocol did not mandate management changes based on MI results. Therefore, treatment intensification was made based on clinician judgment, limiting the interpretation of our results as factors other than the MI alone may have influenced a final decision to intensify treatment or not. We have considered the addition of ADT as an appropriate therapeutic intensification, although there is still controversy about the treatment duration and the selection of patients who can better benefit from ADT plus sRT following BCR [19, 20]. Another limitation was the inability to consider other aspects of treatment intensification such as the duration of hormones or the inclusion of pelvic nodal irradiation. For example, the recently reported NRG 0543 SPPORT trial reported improved outcomes with both addition of hormone therapy as well as incremental benefit of addition of pelvic nodal radiation [21]. The Stephenson nomogram used to predict benefit only considers any use of ADT as a predictive factor, not duration of ADT nor does it include pelvic nodal irradiation.

Moreover, the use of nomograms to predict long-term outcomes as a surrogate for biochemical failure is a limitation. However, for the purpose of this study which aimed to identify if MI could improve patient’s selection for ADT intensification (and not outcome), the integration of several acknowledged prognosis factors by the nomogram into a recurrence risk for each patient is a valid approach and probably one of the most objective since it is not biased by the clinician’s opinion. Finally, we excluded patients for which AS was decided pre- or post- MI because we could not determine if this choice was because lack of response to sRT was expected or because the clinician thought the patient had a clinically insignificant recurrence. This exclusion significantly reduced the change in management plan from 42 to 28% in the final analyses. This could have introduced some bias.

## Conclusions

PSMA PET/CT and/or Choline PET/CT before sRT can potentially improve patient management by directing clinicians towards treatment intensification or de-intensification with ADT. Using a validated predictive nomogram, MI appeared to better stratify patients into groups who would benefit from salvage radiotherapy alone vs. addition of ADT. More prospective studies are needed with a long-term follow-up to determine the best individualized treatment intensification based on MI have an impact on long term patient outcomes and such trials are in progress.

## Electronic supplementary material

Below is the link to the electronic supplementary material.

Supplementary Material 1

Supplementary Material 2

Supplementary Material 3

## Data Availability

The datasets analyzed during the current study are available from the corresponding author upon reasonable request.

## Abbreviations and Acronyms

ADT: Androgen deprivation therapy
AS: Active surveillance
BCR: Biochemical recurrence
BFS: Biochemical free survival
CT: Computed tomography
FFP: Freedom from progression
MI: Molecular Imaging
PCa: Prostate cancer
PSA: Prostate-specific antigen
PSMA: Prostate Specific Membrane Antigen
RP: Radical prostatectomy
sRT: Salvage radiotherapy
