# Supplementary material for: Influence of molecular imaging on patient selection for treatment intensification prior to salvage radiation therapy for prostate cancer: a post hoc analysis of the PROPS trial
Source: Cancer Imaging. 2023 Jun 8;23:57. doi: 10.1186/s40644-023-00570-x (PMC10249189; doi:10.1186/s40644-023-00570-x)
Supplement: Supplementary file 1 — Supplementary Material 1 [file 40644_2023_570_MOESM1_ESM.docx]

**Supplemental Figure 1**. Box plot of the 5-year biochemical free survival (BFS) for salvage radiotherapy (sRT) after radical prostatectomy based on the updated Stephenson **
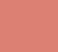

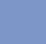
**nomogram.**
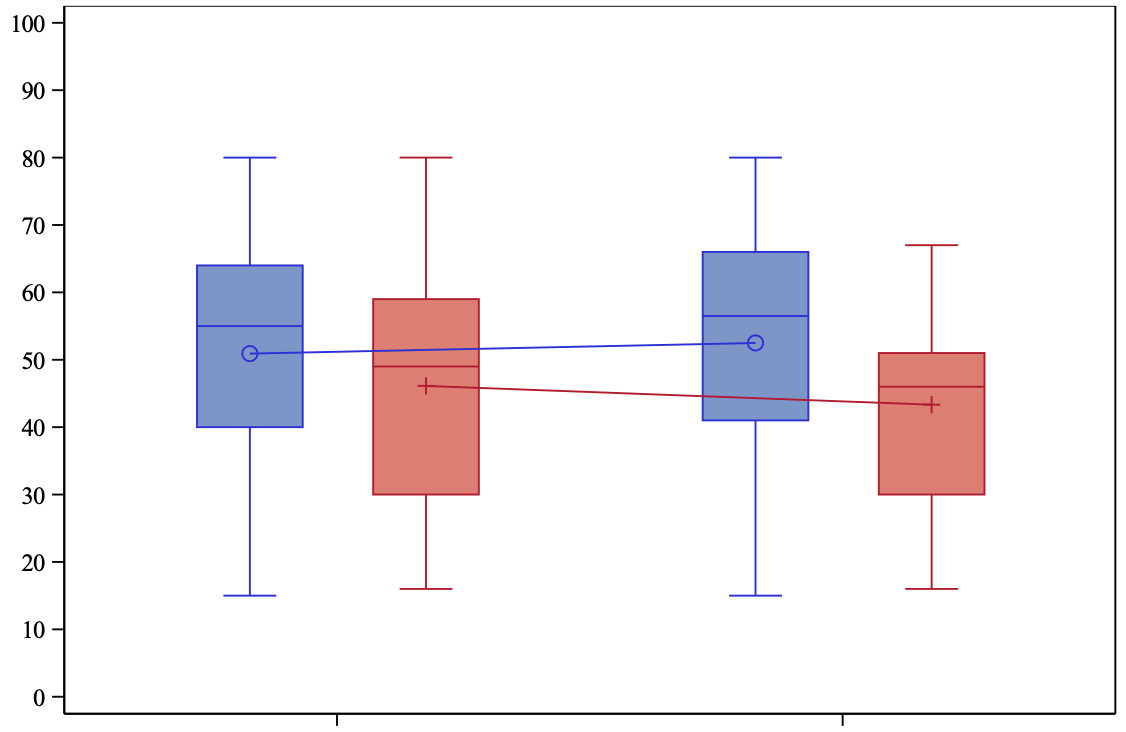
**

Therapeutic decision sRT alone ADT ± sRT

Pre-molecular imaging

Post-molecular imaging

5- years BFS probability (%)
