# Supplementary material for: Influence of molecular imaging on patient selection for treatment intensification prior to salvage radiation therapy for prostate cancer: a post hoc analysis of the PROPS trial
Source: Cancer Imaging. 2023 Jun 8;23:57. doi: 10.1186/s40644-023-00570-x (PMC10249189; doi:10.1186/s40644-023-00570-x)
Supplement: Supplementary file 2 — Supplementary Material 2 [file 40644_2023_570_MOESM2_ESM.docx]

**Influence of Molecular Imaging on Patient Selection for Treatment Intensification Prior to Salvage Radiation Therapy for Prostate Cancer: A Post Hoc Analysis of the PROPS Trial**

Samuel Tremblay^1^, Mofarej Alhogbani^1^, Andrew Weickhardt^2^, Ian D Davis^3^, Andrew M Scott^2^, Rodney J Hicks^4^, Ur Metser^5^, Sue Chua^6^, Reena Davda^7^, Shonit Punwani^8^, Heather Payne^9^, Nina Tunariu^6^,Bao Ho^8^, Sympascho Young^9^, Mahukpe Narcisse Ulrich Singbo^1^, Glenn Bauman^9^, Louise Emmett^8^, Frédéric Pouliot^1^

^1^ CHU de Québec and Université Laval, Québec, Québec, Canada
^2^ Austin Health and University of Melbourne, Olivia Newton-John Cancer Research Institute and La Trobe University, Melbourne, Australia
^3^ Monash University Eastern Health Clinical School, Box Hill, Victoria, Australia
^4^ Peter MacCallum Cancer Centre, Melbourne, Australia
^5^ University of Toronto, Toronto, Ontario, Canada
^6^ Royal Marsden Hospital, London, United Kingdom
^7^ University College London, London, United Kingdom

^8^ St. Vincent’s Hospital, Sydney, New South Wales, Australia
^9^ London Health Sciences Centre, London, Ontario, Canada

**Corresponding Author:**

Frédéric Pouliot, MD, PhD, FRCSC

Uro-oncologist, CHU de Québec-Université Laval

Oncology Axis, CHU de Québec–Université Laval Research Centre, Quebec City, QC, Canada G1V 4G2

Tel: 418-525-4444 ext. 67694

[frederic.pouliot@crchudequebec.ulaval.ca](mailto:frederic.pouliot@crchudequebec.ulaval.ca)

**Abstract word count:** 251 words **Article word count**: 2037 words

**Key words:** Prostate cancer; biochemical recurrence; molecular imaging; PSMA; Choline.

**Abstract**

**Background**

The impact of molecular imaging (MI) on patient management after biochemical recurrence (BCR) following radical prostatectomy has been described in many studies. However, it is not known if MI-induced management changes are appropriate. This study aimed to determine if androgen deprivation therapy (ADT) management plan is improved by MI in patients who are candidates for salvage radiation therapy.

**Methods**

Data were analyzed from the multicenter prospective PROPS trial evaluating PSMA/Choline PET in patients being considered for salvage radiotherapy (sRT) with BCR after prostatectomy. We compared the pre- and post-MI ADT management plans for each patient and cancer outcomes as predicted by the MSKCC nomogram. A higher percentage of predicted BCR associated with ADT treatment intensification after MI was considered as an improvement in a patient’s management.

**Results**

Seventy-three patients with a median PSA of 0.38 ng/mL were included. In bivariate analysis, a positive finding on MI (local or metastatic) was associated with decision to use ADT with an odds ratio of 3.67 (95% CI, 1.25 to 10.71; p=0.02). No factor included in the nomogram was associated with decision to use ADT. Also, MI improved selection of patients to receive ADT based on predicted BCR after sRT : the predicted nomogram 5-year biochemical-free survivals were 52.5 % and 43.3%, (mean difference, 9.2%; 95% CI 0.8 to 17.6; p=0.03) for sRT alone and ADT±sRT subgroups, while there was no statistically significant difference between subgroups before MI.

**Conclusions**

PSMA and/or Choline PET/CT before sRT can potentially improve patient ADT management by directing clinicians towards more appropriate intensification.

**INTRODUCTION**

Prostate cancer (PCa) is the second most frequently diagnosed cancer and the fifth cause of cancer death in men worldwide (1). Primary PCa locoregional therapies are mainly radical prostatectomy (RP) and radiotherapy. Unfortunately, biochemical recurrence (BCR) can occur in up to 40% after 10-year following RP (2, 3). Following BCR, conventional imaging such as computed tomography (CT) and bone scintigraphy lack sensitivity for the detection of local or metastatic recurrences at a low level of prostate-specific antigen (PSA) values (4).

There is a growing role for molecular imaging (MI) targeting Prostate Specific Membrane Antigen (PSMA), Choline metabolism, or amino acid transporter using specific tracers. These novel approaches have been shown to be significantly more accurate than conventional imaging for the detection of PCa metastasis, especially after primary therapies and at low PSA values (5-8). These new imaging modalities are now considered a standard of care for re-staging patients after RP (9). While many studies have reported how MI can impact the management of PCa, few prospective studies reported if the change in management induced by MI improved the patient’s outcomes (10).

The PROPS trial was conducted to evaluate the detection rate of lesion by PSMA-PET/CT and/or Choline-PET/CT in patients who were considered for salvage radiotherapy (sRT) after RP and who had negative or equivocal conventional imaging (8, 11). The PROPS trial showed that these MI technologies had an important impact on the management plan for clinicians, leading to a management change in 42% of cases (8). However, it is unknown if these management changes improve patient care. In this study, we seek to evaluate if PSMA-PET/CT or Choline-PET/CT MI improves patient selection for treatment intensification with ADT in patients suitable for sRT, based on a predictive nomogram.

**MATERIALS AND METHODS**

Full materials and methods of the PROPS trial have been previously published (8, 11). Briefly, the PROPS trial is a prospective, international multicenter trial including men with features of high-risk prostate cancer being considered for sRT and who had PSA recurrence after RP. The study protocol was approved by all institutional ethics board and registered on clinicaltrials.gov (NCT02131649). Initial inclusion criteria were : patients with biopsy confirmed PCa prior RP for pT1-pT3, N0/Nx disease, a rising PSA level of at least 0.2 ng/mL (3 consecutive rises documented a minimum of 2 weeks apart), and at least one high-risk feature (PSA >1 ng/mL, ≥ pT3b, Gleason score >7, or PSA doubling time ≤ 10 months). All patients included had a negative or equivocal diagnostic CT and bone scan 12 weeks before enrollment and had a planned management of standard sRT with curative intent. Patients underwent PSMA-PET/CT and/or Choline-PET/CT, and pelvic multiparametric pelvic or whole-body MRI to identify potential recurrence location. All treating physicians were asked to undertake a pre-imaging questionnaire to document their intended management, including planned sRT site, doses and fractions of radiotherapy, and ADT duration if it was planned. A second questionnaire was completed after imaging to document management changes. The study did not dictate the treatments to be received, but were all documented. The study provided for three-year follow-up of patients allowing for assessment of short term clinical endpoints (such as PSA response post treatment) but not long term outcomes (such as long term biochemical control, disease free survival, metastases free survival or overall survival).

For the current study, management plans were analyzed pre- and post-imaging for every patient initially included in the PROPS trial. Patients were evaluated based on treatment decisions before and after MI. The addition of ADT to sRT or ADT alone (ADT±sRT) was considered as a treatment intensification. Patients managed by active surveillance (AS) after new imaging modalities were excluded from final analyses because we considered that these patients could have been put on AS for aggressive (not responsive to sRT) or indolent disease (not benefiting from sRT). A detailed consort diagram summarizing included patients for this study is shown in Figure 1. As a proxy for long term outcomes, the updated Stephenson et al. predictive nomogram for sRT after RP was used to evaluate the predicted 5- and 10-year biochemical-free survival according to patient clinicopathological characteristics (12). Criteria for the nomogram were: surgical Gleason Grade (≤6, 7, 8, 9-10), extraprostatic extension (Yes/No), surgical margin status (Positive/Negative), seminal vesicle invasion (Yes/No), pre-sRT PSA level (ng/mL) and prostate bed radiation dose (≥ 6600 or <6600 cGy) and use of ADT with sRT. Projected outcome for every patient was calculated assuming sRT alone (no neoadjuvant or concurrent ADT) as the baseline (pre-MI) treatment plan.

The primary endpoint of this study was the percentage of BCR predicted by the Stephenson nomogram before and after MI. A higher percentage of BCR associated with treatment intensification (ADT±sRT) after MI was considered as improved management.

**Statistical Analysis**

Analytical statistics were generated by SAS 9.4 Software. Bivariate logistic regression with Wald-based confidence intervals was used to correlate the association between imaging findings, Stephenson’s criteria and treatment decision. The comparison of mean predicted outcomes were made using two-sample Student t-tests. All tests were 2-sided, and a *p*-value of 0.05 or less was considered statistically significant.

**RESULTS**

Ninety patients were included in the PROPS Trial and the management plan was changed by the treating physician in 42% of patients (38/90) after re-imaging by PSMA PET/CT and/or choline PET/CT. Of the 38 patients that had a change in management after MI, 6 (7%) had ADT de-intensification from ADT+sRT to sRT alone, 15 patients (17%) had ADT intensification (prescription of ADT alone or in combination to sRT from either pre-imaging surveillance or sRT alone) and 17 (19%) had ADT de-intensification to surveillance and were excluded from final analyses. All patients were imaged with Choline PET/CT, and 23 patients (32%) were also imaged with PSMA PET/CT. Patient characteristics are summarized in table 1.

We first assessed if MI influenced clinicians in the decision to intensify therapy. We found that a positive finding on MI, whether local or metastatic, was associated with a decision to prescribe ADT (Table 2, odds ratio = 3.67; 95% CI, 1.25 to 10.71; p=0.02). If a metastatic lesion was found on MI, the odds ratio increased to 3.83 (95% CI, 1.11 to 13.30; p=0.03). Interestingly, none of the Stephenson nomogram criteria used separately was associated with a decision to prescribe ADT based on the initial study cohort (Table 3). Patient’s characteristics from the initial study are summarized in the supplementary Table 1.

To determine the appropriateness of ADT intensification or de-intensification, we raised the hypothesis that an appropriate ADT management change induced by MI would intensify ADT prescription in patients with worst nomogram predicted prognosis, and de-intensify ADT prescription in patients with better nomogram predicted prognosis. To determine the risk of recurrence for each patient, the Stephenson nomogram was used (12). Before MI, the predicted mean 5-year biochemical-free survival was 50.9% and 46.1% (mean difference, 4.8%; 95% CI -4.6 to 14.2) for sRT alone and ADT±sRT subgroups, respectively (p=0.3). After MI, the predicted mean 5-year biochemical-free survival was 52.5 % and 43.3%, (mean difference, 9.2%; 95% CI 0.8 to 17.6) for sRT alone and ADT±sRT subgroups, respectively (p=0.03). Indeed, the difference between the two subgroups in predicted 5-year biochemical-free survival almost doubled after MI, increasing to 9.2% from 4.8% (Supplemental Figure 1). The predicted 10-year biochemical-free survival mean difference was also statistically significant between subgroups after MI (9.7%; 95% CI 1.9 to 18.2, p=0.03) while it was not before (Table 4).

**DISCUSSION**

In recent years, MI use has been increasingly adopted into clinical practice. These new imaging modalities have clearly demonstrated an increase in the accuracy for identifying metastasis when compared to conventional imaging, leading to a shift towards treatment intensification, either by targeting oligometastatic disease and/or by adding ADT. The benefits of treatment intensification in patients with recurrent PCa after RP is poorly studied and mostly based on the extrapolation of small studies performed in metastatic patients proven by conventional imaging (13, 14). So far, no prospective muticenter study has evaluated long-term prognostic outcomes of these patients diagnosed early with MI.

With 42% management changes observed after MI, our study compares to recent literature (15-17). Here we were able to demonstrate that a positive finding on MI prompted clinicians to intensify therapy, at least with ADT. Interestingly, clinicians tended to intensify therapy if a lesion was detected, no matter if it was in the prostate bed or as a metastasis. This means that clinicians appear to be influenced by MI findings in their therapeutic decision-making and consider both local and metastatic lesions as being more aggressive.

We also demonstrated that the therapeutic intensification induced by MI was appropriate for this cohort of patients. Using the predictive Stephenson nomogram, we modeled a statistically significant biochemical free-survival difference between patients with MI directed therapeutic intensification (ADT±sRT subgroup) management plans compared to the projected results with sRT alone at 5- and 10-years.
These results suggest that MI can differentiate patients with a poorer prognosis who might better benefit from therapeutic intensification. Therefore, MI may integrate many poor prognostic factors as an imageable lesion that guides the clinicians toward identification of more aggressive disease.

Literature on the benefits of treatment changes after MI is limited. The EMPIRE-1 study showed promising results with ^18^F-fluciclovine-PET/CT. One hundred sixty-five patients with negative conventional imaging were randomized to sRT directed by conventional imaging alone or to conventional plus ^18^F-fluciclovine-PET/CT. Three-year event-free survival was significantly superior to the ^18^F-fluciclovine-PET/CT group (75.5% vs 63.0%, *p*=0.0028). Importantly, toxicity was similar in both groups (10). Emmett et al. also demonstrated interesting results with PSMA-PET/CT. Two hundred sixty patients with a rising PSA level after RP were prospectively referred to PSMA-PET/CT. Three years’ freedom from progression (FFP) after sRT was highly predictive with a reported FFP of 64.5% (120/186) overall, 81% (81/100) with fossa-confined disease and 45% (39/86) with extrafossa disease after PSMA-PET/CT (18). These results and ours are supporting that MI rationally helps to locate disease recurrence and select patients that could benefit from a treatment intensification and potentially impact oncologic outcomes. The Ongoing PATRON trial (NCT04557501) and PSMA-SRT trial (NCT03582774) are two randomized Phase III studies that will evaluate cancer outcomes of treatment intensification with PSMA PET/CT compared to conventional imaging on a 5-year perspective and will certainly help to clarify how to manage these oligometastatic patients.

There are limitations to our study. The original protocol did not mandate management changes based on MI results. Therefore, treatment intensification was made based on clinician judgment, limiting the interpretation of our results as factors other than the MI alone may have influenced a final decision to intensify treatment or not. We have considered the addition of ADT as an appropriate therapeutic intensification, although there is still controversy about the treatment duration and the selection of patients who can better benefit from ADT plus sRT following BCR (19, 20). Another limitation was the inability to consider other aspects of treatment intensification such as the duration of hormones or the inclusion of pelvic nodal irradiation. For example, the recently reported NRG 0543 SPPORT trial reported improved outcomes with both addition of hormone therapy as well as incremental benefit of addition of pelvic nodal radiation (21). The Stephenson nomogram used to predict benefit only considers any use of ADT as a predictive factor, not duration of ADT nor does it include pelvic nodal irradiation.

Moreover, the use of nomograms to predict long-term outcomes as a surrogate for biochemical failure is a limitation. However, for the purpose of this study which aimed to identify if MI could improve patient’s selection for ADT intensification (and not outcome), the integration of several acknowledged prognosis factors by the nomogram into a recurrence risk for each patient is a valid approach and probably one of the most objective since it is not biased by the clinician’s opinion. Finally, we excluded patients for which AS was decided pre- or post- MI because we could not determine if this choice was because lack of response to sRT was expected or because the clinician thought the patient had a clinically insignificant recurrence. This exclusion significantly reduced the change in management plan from 42% to 28% in the final analyses. This could have introduced some bias.

**CONCLUSIONS**

PSMA PET/CT and/or Choline PET/CT before sRT can potentially improve patient management by directing clinicians towards treatment intensification or de-intensification with ADT. Using a validated predictive nomogram, MI appeared to better stratify patients into groups who would benefit from salvage radiotherapy alone vs. addition of ADT. More prospective studies are needed with a long-term follow-up to determine the best individualized treatment intensification based on MI have an impact on long term patient outcomes and such trials are in progress.

**ABBREVIATIONS AND ACRONYMS**

ADT : Androgen deprivation therapy

AS : active surveillance

BCR : Biochemical recurrence

BFS : Biochemical free survival

CT : Computed tomography

FFP : Freedom from progression

MI: Molecular Imaging

PCa : Prostate cancer

PSA : Prostate-specific antigen

PSMA : Prostate Specific Membrane Antigen

RP : Radical prostatectomy

sRT : salvage radiotherapy

**DECLARATIONS**

**Ethics approval and consent to participate**

This research project has been approved by the *Comité d’éthique de la recherche du CHU de Québec – Université Laval* (Number 2014-1928)

**Consent for publication**

Not applicable.

**Availability of data and materials**

The datasets analyzed during the current study are available from the corresponding author upon reasonable request.

**Competing interests**

The authors declare that they have no competing interests.

**Funding**

This work was funded by Movember as part of the Global Action Plan (GAP2) Initiative.

**Authors' contributions**

FP contributed to the conception and design of the study, analysis of results, drafting of the manuscript and substantively revised it. ST contributed to data management, analysis of results and drafting of the manuscript. MA contributed to the collection of the data. MNUS contributed to data management and analysis of results. AW, IDD, AMS, RJH, UM, SC, RD, SP, HP, NT, BH, SY, GB, LE substantively revised the manuscript. All authors read and approved the final manuscript.

**Acknowledgements**

The authors would like to thank Movember’s many fundraisers and donors from around the world for their contributions.

**Figure 1.** Consort diagram summarizing patients included in our analysis. Salvage radiotherapy (sRT); Androgen deprivation therapy (ADT); Active surveillance (AS).

Patients evaluated with **negative/equivocal** conventional imaging included in the

PROPS trial

(n = 90)

sRT alone

(n = 52)

ADT + sRT or ADT alone

(n = 21)

Management plan **before** molecular PET/CT

(n = 73)

sRT alone

(n = 57)

ADT + sRT

(n = 16)

Management plan **after** molecular PET/CT

(n = 73)

Excluded as they were managed by AS

(n = 17)

**Table 1.** Patients characteristics

| **Characteristics** | **Value** |
| --- | --- |
| Number of patients | *N* = 73 |
| Surgical Gleason score no. (%)   - 6 - 7 - 8 - 9 | 1 (1.4)  52 (71.2)  11 (15.1)  9 (12.13) |
| Positive surgical margins no. (%) | 45 (61.6) |
| Extracapsular Extension no. (%) | 25 (34.2) |
| Seminal Vesicle Invasion no. (%) | 16 (21.9) |
| Pre-sRT PSA Level ng/ml median. (range) | 0.38 (0.29-0.93) |

**Table 2.** Association between imaging findings location with PSMA- and/or Choline-PET/CT and treatment decision. Salvage radiotherapy (sRT); Androgen deprivation therapy (ADT).

|  |  | **Therapeutic decision** | | | |  |  |
| --- | --- | --- | --- | --- | --- | --- | --- |
|  |  | **sRT** | | **ADT ± sRT** | |  |  |
|  | **Location** | **n/N** | **%** | **n/N** | **%** | **Odds Ratio (95% CI)** | **P-value^1^** |
| **Molecular imaging findings** | Extraprostatic  Fossa  Negative | 6/13  6/10  40/50 | 46.2  60.0  80.0 | 7/13  4/10  10/50 | 53.8  40.0  20.0 | 4.67(1.28;16.99)  2.67(0.63;11.28) | 0.0490* |
|  | Positive  Negative | 12/23  40/50 | 52.2  80.0 | 11/23  10/50 | 47.8  20.0 | 3.67(1.25;10.71) | 0.0175* |
|  | Metastatic  Non-metastatic | 6/13  46/60 | 46.2  76.7 | 7/13  14/60 | 53.8  23.3 | 3.83(1.11;13.30) | 0.0342* |

^1^Based on Bivariate Logistic Regression with Wald confidence interval, and Firth Correction for bias (C) when

appropriate. * <0.05

**Table 3.** Stephenson nomogram criteria as predictors of therapeutic change after molecular imaging based on the whole study cohort. Salvage radiotherapy (sRT); Androgen deprivation therapy (ADT).

|  | **Therapeutic decision** | **n** | **Mean** | **SD** | **95% CI** | **Odds Ratio (95% CI)** | **P-value^1^** |
| --- | --- | --- | --- | --- | --- | --- | --- |
| **Pre-sRT PSA** | sRT  ADT ± sRT | 71  19 | 0.39  0.63 | 0.49  0.50 | (0.28; 0.51)  (0.39; 0.87) | 2.63(0.92;7.50) | 0.0699^T^ |
| **Surgical Gleason score** | sRT  ADT ± sRT | 71  20 | 7.42  7.50 | 0.71  0.89 | (7.25; 7.59)  (7.08; 7.92) | 1.15(0.60;2.19) | 0.6814 |
|  |  | **Therapeutic decision** | | | |  |  |
|  |  | **sRT** | | **ADT ± sRT** | |  |  |
|  |  | **n/N** | **%** | **n/N** | **%** | **Odds Ratio (95% CI)** | **P-value^1^** |
| **Extraprostatic extention** | No  Yes | 28/71  43/71 | 39.4  60.6 | 6/20  14/20 | 30.0  70.0 | 1.52(0.52;4.42) | 0.4429 |
| **Surgical margins** | Negatives  Positives | 51/71  20/71 | 71.8  28.2 | 13/20  7/20 | 65.0  35.0 | 1.37(0.48;3.94) | 0.5556 |
| **Seminal vesicle invasion** | No  Yes | 55/71  16/71 | 77.5  22.5 | 14/20  6/20 | 70.0  30.0 | 0.68(0.22;2.05) | 0.4924 |

^1^Based on Bivariate Logistic Regression with Wald confidence interval, and Firth Correction for bias (C)

when appropriate. T < 0.15

**Table 4.** Predicted 5- and 10-year biochemical free survival (BFS) for salvage radiotherapy (sRT) after radical prostatectomy based on the updated Stephenson nomogram.

|  |  | **Therapeutic decision** | |  |  |
| --- | --- | --- | --- | --- | --- |
|  |  | **sRT alone (%)** | **ADT ± sRT (%)** | **Mean Difference (95% CI)** | **P-value^1^** |
| **Pre-molecular imaging** | 5-year BFS probability | 50.91 | 46.13 | 4.79 (-4.63;14.20) | 0.3141 |
|  | 10-year BFS probability | 38.16 | 33.56 | 4.60 (-4.99;14.18) | 0.3423 |
| **Post-molecular imaging** | 5-year BFS probability | 52.5 | 43.3 | 9.2 (0.78;17.56) | 0.0327* |
|  | 10-year BFS probability | 39.94 | 30.24 | 9.70 (1.93;18.22) | 0.0260* |

^1^ Based on two-sample Student’s t-Tests
 * <0.05**Supplemental Figure 1**. Box plot of the 5-year biochemical free survival (BFS) for salvage radiotherapy (sRT) after radical prostatectomy based on the updated Stephenson **
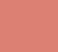

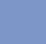
**nomogram.**
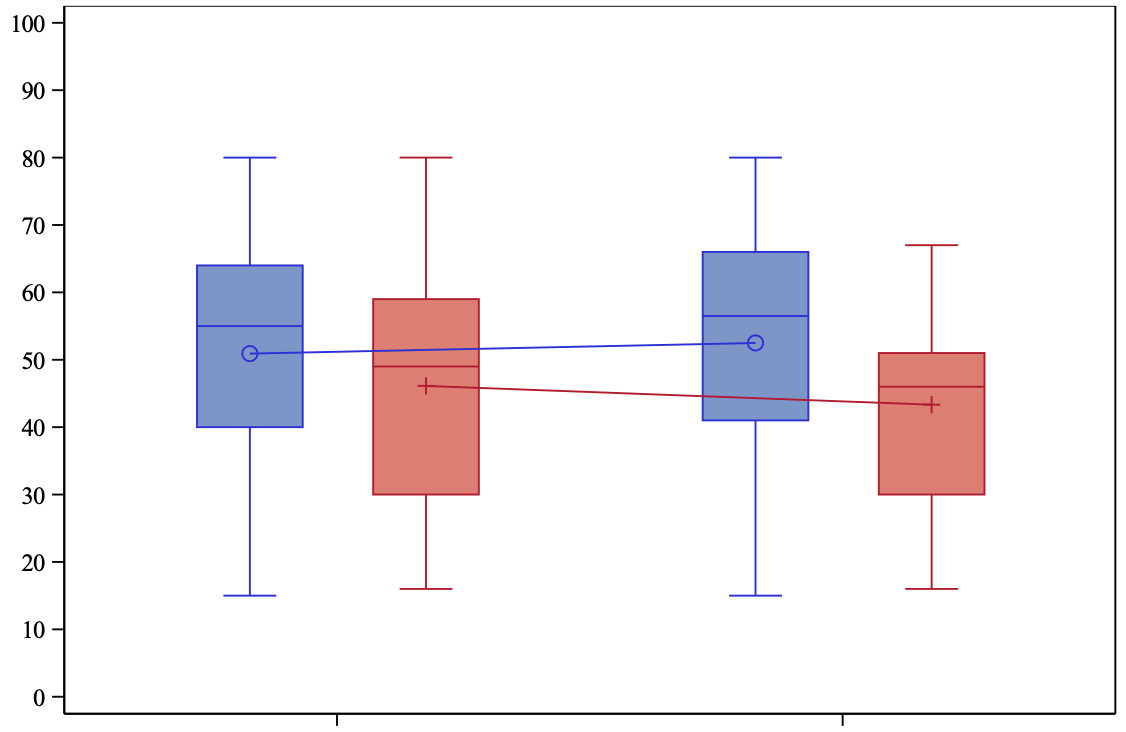
**

Therapeutic decision sRT alone ADT ± sRT

Pre-molecular imaging

Post-molecular imaging

5- years BFS probability (%)

**Supplemtary table 1.** Patients characteristics from the whole study cohort

| **Characteristics** | **Value** |
| --- | --- |
| Number of patients | *N* = 90 |
| Surgical Gleason score no. (%)   - 6 - 7 - 8 - 9 | 1 (1.1)  62 (68.9)  14 (15.6)  13 (14.4) |
| Positive surgical margins no. (%) | 47 (52.2) |
| Extracapsular Extension no. (%) | 32 (35.6) |
| Seminal Vesicle Invasion no. (%) | 22 (24.4) |
| Pre-sRT PSA Level ng/ml median. (range) | 0.42 (0.29-0.93) |

**REFERENCES**

1. Sung H, Ferlay J, Siegel RL, Laversanne M, Soerjomataram I, Jemal A, et al. Global Cancer Statistics 2020: GLOBOCAN Estimates of Incidence and Mortality Worldwide for 36 Cancers in 185 Countries. CA Cancer J Clin. 2021;71(3):209-49.

2. Roehl KA, Han M, Ramos CG, Antenor JA, Catalona WJ. Cancer progression and survival rates following anatomical radical retropubic prostatectomy in 3,478 consecutive patients: long-term results. J Urol. 2004;172(3):910-4.

3. Freedland SJ, Humphreys EB, Mangold LA, Eisenberger M, Dorey FJ, Walsh PC, et al. Risk of prostate cancer-specific mortality following biochemical recurrence after radical prostatectomy. Jama. 2005;294(4):433-9.

4. Beresford MJ, Gillatt D, Benson RJ, Ajithkumar T. A systematic review of the role of imaging before salvage radiotherapy for post-prostatectomy biochemical recurrence. Clin Oncol (R Coll Radiol). 2010;22(1):46-55.

5. Fuccio C, Castellucci P, Schiavina R, Guidalotti PL, Gavaruzzi G, Montini GC, et al. Role of 11C-choline PET/CT in the re-staging of prostate cancer patients with biochemical relapse and negative results at bone scintigraphy. Eur J Radiol. 2012;81(8):e893-6.

6. Giovacchini G, Picchio M, Briganti A, Cozzarini C, Scattoni V, Salonia A, et al. [11C]choline positron emission tomography/computerized tomography to restage prostate cancer cases with biochemical failure after radical prostatectomy and no disease evidence on conventional imaging. J Urol. 2010;184(3):938-43.

7. Grubmüller B, Baltzer P, D'Andrea D, Korn S, Haug AR, Hacker M, et al. (68)Ga-PSMA 11 ligand PET imaging in patients with biochemical recurrence after radical prostatectomy - diagnostic performance and impact on therapeutic decision-making. Eur J Nucl Med Mol Imaging. 2018;45(2):235-42.

8. Emmett L, Metser U, Bauman G, Hicks RJ, Weickhardt A, Davis ID, et al. Prospective, Multisite, International Comparison of (18)F-Fluoromethylcholine PET/CT, Multiparametric MRI, and (68)Ga-HBED-CC PSMA-11 PET/CT in Men with High-Risk Features and Biochemical Failure After Radical Prostatectomy: Clinical Performance and Patient Outcomes. J Nucl Med. 2019;60(6):794-800.

9. Cornford P, van den Bergh RCN, Briers E, Van den Broeck T, Cumberbatch MG, De Santis M, et al. EAU-EANM-ESTRO-ESUR-SIOG Guidelines on Prostate Cancer. Part II-2020 Update: Treatment of Relapsing and Metastatic Prostate Cancer. Eur Urol. 2021;79(2):263-82.

10. Jani AB, Schreibmann E, Goyal S, Halkar R, Hershatter B, Rossi PJ, et al. (18)F-fluciclovine-PET/CT imaging versus conventional imaging alone to guide postprostatectomy salvage radiotherapy for prostate cancer (EMPIRE-1): a single centre, open-label, phase 2/3 randomised controlled trial. Lancet. 2021;397(10288):1895-904.

11. Metser U, Chua S, Ho B, Punwani S, Johnston E, Pouliot F, et al. The Contribution of Multiparametric Pelvic and Whole-Body MRI to Interpretation of (18)F-Fluoromethylcholine or (68)Ga-HBED-CC PSMA-11 PET/CT in Patients with Biochemical Failure After Radical Prostatectomy. J Nucl Med. 2019;60(9):1253-8.

12. Tendulkar RD, Agrawal S, Gao T, Efstathiou JA, Pisansky TM, Michalski JM, et al. Contemporary Update of a Multi-Institutional Predictive Nomogram for Salvage Radiotherapy After Radical Prostatectomy. J Clin Oncol. 2016;34(30):3648-54.

13. Phillips R, Shi WY, Deek M, Radwan N, Lim SJ, Antonarakis ES, et al. Outcomes of Observation vs Stereotactic Ablative Radiation for Oligometastatic Prostate Cancer: The ORIOLE Phase 2 Randomized Clinical Trial. JAMA Oncol. 2020;6(5):650-9.

14. Palma DA, Olson R, Harrow S, Gaede S, Louie AV, Haasbeek C, et al. Stereotactic ablative radiotherapy versus standard of care palliative treatment in patients with oligometastatic cancers (SABR-COMET): a randomised, phase 2, open-label trial. Lancet. 2019;393(10185):2051-8.

15. Han S, Woo S, Kim YJ, Suh CH. Impact of (68)Ga-PSMA PET on the Management of Patients with Prostate Cancer: A Systematic Review and Meta-analysis. Eur Urol. 2018;74(2):179-90.

16. Andriole GL, Kostakoglu L, Chau A, Duan F, Mahmood U, Mankoff DA, et al. The Impact of Positron Emission Tomography with 18F-Fluciclovine on the Treatment of Biochemical Recurrence of Prostate Cancer: Results from the LOCATE Trial. J Urol. 2019;201(2):322-31.

17. Morris MJ, Rowe SP, Gorin MA, Saperstein L, Pouliot F, Josephson D, et al. Diagnostic Performance of (18)F-DCFPyL-PET/CT in Men with Biochemically Recurrent Prostate Cancer: Results from the CONDOR Phase III, Multicenter Study. Clin Cancer Res. 2021;27(13):3674-82.

18. Emmett L, Tang R, Nandurkar R, Hruby G, Roach P, Watts JA, et al. 3-Year Freedom from Progression After (68)Ga-PSMA PET/CT-Triaged Management in Men with Biochemical Recurrence After Radical Prostatectomy: Results of a Prospective Multicenter Trial. J Nucl Med. 2020;61(6):866-72.

19. Carrie C, Magné N, Burban-Provost P, Sargos P, Latorzeff I, Lagrange JL, et al. Short-term androgen deprivation therapy combined with radiotherapy as salvage treatment after radical prostatectomy for prostate cancer (GETUG-AFU 16): a 112-month follow-up of a phase 3, randomised trial. Lancet Oncol. 2019;20(12):1740-9.

20. Shipley WU, Seiferheld W, Lukka HR, Major PP, Heney NM, Grignon DJ, et al. Radiation with or without Antiandrogen Therapy in Recurrent Prostate Cancer. N Engl J Med. 2017;376(5):417-28.

21. Pollack A, Karrison TG, Balogh AG, Gomella LG, Low DA, Bruner DW, et al. The addition of androgen deprivation therapy and pelvic lymph node treatment to prostate bed salvage radiotherapy (NRG Oncology/RTOG 0534 SPPORT): an international, multicentre, randomised phase 3 trial. Lancet. 2022;399(10338):1886-901.
