# Supplementary material for: Influence of molecular imaging on patient selection for treatment intensification prior to salvage radiation therapy for prostate cancer: a post hoc analysis of the PROPS trial
Source: Cancer Imaging. 2023 Jun 8;23:57. doi: 10.1186/s40644-023-00570-x (PMC10249189; doi:10.1186/s40644-023-00570-x)
Supplement: Supplementary file 3 — Supplementary Material 3 [file 40644_2023_570_MOESM3_ESM.docx]

**Supplementary Table 1.** Patient’s characteristics from the whole study cohort

| **Characteristics** | **Value** |
| --- | --- |
| Number of patients | *N* = 90 |
| Surgical Gleason score no. (%)   - 6 - 7 - 8 - 9 | 1 (1.1)  62 (68.9)  14 (15.6)  13 (14.4) |
| Positive surgical margins no. (%) | 47 (52.2) |
| Extracapsular Extension no. (%) | 32 (35.6) |
| Seminal Vesicle Invasion no. (%) | 22 (24.4) |
| Pre-sRT PSA Level ng/ml median. (range) | 0.42 (0.29-0.93) |
